# Quantitative Determination of Salivary Cariogenic Bacteria in Patients With Fixed Orthodontic Appliances

**DOI:** 10.7759/cureus.71290

**Published:** 2024-10-12

**Authors:** Fighan J Hussein, Maryam F Bilal, Tara S Hamad, Rwa Al-Qazzaz

**Affiliations:** 1 Department of Pedodontics, Orthodontics, and Preventive Dentistry, College of Dentistry, Hawler Medical University, Erbil, IRQ

**Keywords:** lactobacillus, orthodontic fixed, plaque, real-time pcr, saliva, streptococcus mutans

## Abstract

Background and objectives: Fixed orthodontic treatment can alter the oral environment, potentially increasing cariogenic bacteria levels. This study aimed to utilize real-time polymerase chain reaction (qPCR) to quantify salivary levels of cariogenic bacteria in patients undergoing orthodontic treatment with fixed appliances.

Methods: This case-control study was conducted in Erbil, Iraq, from April 2024 to August 2024. One hundred participants were included in the study by random sampling method and equally divided into two groups: case group or orthodontic group and control group. The orthodontic group included participants with fixed orthodontic brackets, while the control group included participants without current or past orthodontic treatment with matching age and sex as the orthodontic group. Data were collected via questionnaires, clinical examinations, and saliva sampling. Salivary samples were analyzed using real-time PCR to detect *Streptococcus mutans* (SM) and *Lactobacillus* (LB) spp. levels.

Results: The age ranged from 18 to 30 years, and the mean age of participants was 23.96 ± 3.64 years. No significant differences were observed between the two groups across most parameters, except in the use of fluoride therapy, where the control group demonstrated better usage (p≤0.003). Real-time PCR results indicated a significant difference in the levels of LB and SM between patients with fixed orthodontics and the control group (p≤0.001). However, no significant differences were found in decay indices such as decayed, missing, and filled teeth; decayed, missing, and filled surfaces; and plaque index in this study.

Conclusions: The quantity and levels of LB and SM were higher in the orthodontic group compared to the control group. Despite the elevated bacterial levels in the orthodontic group, there was no significant difference in decay and plaque indices between the two groups.

## Introduction

Dental caries, one of the most prevalent health issues in the population, is caused by several risk factors, including food, oral hygiene, dental biofilm (also called dental plaque), socioeconomic level, and linked genetic abnormalities, including molar/incisor hypomineralization [1]. The ecological plaque theory posits that an imbalance in the oral cavity is the main factor behind the development of caries. This occurs when caries-causing bacteria multiply while helpful bacteria decline, leading to a shift in dental plaque from a non-cariogenic state to a cariogenic one [2].

Orthodontic treatment using fixed appliances is a prevalent method for correcting dental malocclusion and enhancing oral aesthetics [3]. Despite its effectiveness, this treatment modality poses significant challenges in maintaining oral hygiene. Fixed orthodontic appliances create niches that are difficult to clean, thereby facilitating the accumulation of dental plaque and fostering the proliferation of cariogenic bacteria, such as *Streptococcus mutans* (SM) and *Lactobacillus* (LB) species [4]. Research supports the idea that the use of fixed orthodontic equipment in dental patients might affect the composition of oral microbial communities. For instance, Al-Melh et al. [5] showed that the use of orthodontic brackets results in elevated levels of cariogenic bacteria in saliva. Another study by Thanetchaloempong et al. [1] revealed that the fixed orthodontic caused alterations in the environment and increased the expression of genes associated with tooth decay in the dental biofilm. Hence, understanding the alterations in bacterial composition among patients who had orthodontic fixed treatment has great importance [6].

Various techniques have been used to detect the presence of SM and LB in saliva. In recent times, real-time polymerase chain reaction (qPCR) has emerged as a faster and more sensitive technique for measuring and identifying certain bacterial species [7]. The qPCR technique is capable of accurately detecting the exact quantities of certain bacteria. This method has several uses, including amplification, measurement, and quantification, which may all be performed concurrently to reduce the risk of contamination [6].

Despite the advancements in molecular techniques, there remains a significant gap in the literature regarding the application of real-time qPCR for monitoring cariogenic bacteria in orthodontic patients. Most existing studies rely on conventional methods, which do not provide real-time data on bacterial load. Therefore, the novelty and necessity of the present study lie in its application of real-time qPCR to quantify salivary levels of cariogenic bacteria in patients with fixed orthodontic appliances in Erbil, Iraq. Hence, the present study aimed to utilize real-time qPCR to quantify the salivary levels of cariogenic bacteria in patients undergoing orthodontic treatment with fixed appliances.

## Materials and methods

Study design and setting

This research had a total of 100 individuals, evenly distributed into two groups. The case group or orthodontic group consisted of 50 orthodontic patients who started treatment with fixed orthodontic brackets within the same month, while the control group comprised 50 participants without current or past orthodontic treatment with matching age and sex as the orthodontic group.

The sample size was determined by referencing existing literature and using the Power and Sample Size Calculation version 3.0.43 software (Vanderbilt University Department of Biostatistics, Nashville, TN, USA). The significance level (alpha) was set at 0.05, and the desired statistical power was set at 90%. Accordingly, the estimated sample size was 41 in each group, but a convenience sample of 50 patients wearing fixed orthodontic brackets was taken in the present study, so the whole sample size was 100 subjects.

The inclusion criteria consisted of patients who had intact maxillary and mandibular teeth and had been using fixed orthodontic equipment for a minimum of 12 months. Additionally, they were required to have provided informed permission and be between the ages of 18 and 30. The exclusion criteria included those with gingival inflammation, tooth loss, prosthetic crowns, antibiotic treatment, a history of chemo/radiotherapy to the head and neck, tobacco use, alcohol intake, mouthwash use, recent oral prophylaxis, and difficult behavior. Additionally, the research excluded those with systemic disorders, those who were taking systemic medication, and pregnant women.

Bracket bonding procedure

The fixed orthodontic equipment consisted of a 0.022-in-slot Roth bracket system, which was positioned on the outside surfaces of the upper and lower permanent teeth until the first molars, which were fitted with bondable tubes. Before bonding, the teeth underwent a cleaning process including the application of pumice paste, followed by meticulous rinsing and drying. Isolation was accomplished by using cheek retractors, cotton rollers, and saliva ejectors. The teeth that were going to have brackets attached were treated with 35% phosphoric acid (Ultra-Etch, Ultradent Products Inc., South Jordan, UT, USA) for 30 seconds to etch the enamel. Following a 10-second rinse with water and thorough drying until the enamel exhibited a chalky white appearance, a bonding agent (3M Unitek, 3M, Saint Paul, MN, USA) was applied and then exposed to light for 10 seconds to initiate curing. Subsequently, each bracket was affixed using Transbond Plus Light Cure Orthodontic Adhesive (3M Unitek, 3M, Saint Paul, MN, USA) and subjected to a 20-second light-curing process.

Clinical parameters and sample collection

The study involved three steps: a questionnaire, clinical examination, and saliva sampling.

Questionnaire

Participants were interviewed using a standardized structured questionnaire adapted from Thanetchaloempong et al. to record oral hygiene practices, fluoride use, and dietary sugar habits. The questionnaire was translated into Kurdish and Arabic and verbally explained to illiterate participants. Responses were scored, with correct answers receiving 1 or 2 points and incorrect answers receiving 0 points. Oral hygiene practices were assessed through eight questions, with practice scores categorized as poor (1-7) or good (8-14) [1].

Clinical Examination

Participants underwent a clinical examination under artificial light while seated in a dental chair. The examination assessed plaque and caries using the DMF index, which counts decayed (D), missing (M), and filled (F) teeth (T) or surfaces (S), and the plaque index (PI) was evaluated using Silness and Loe’s scale, measuring plaque on the cervical portion of six teeth (16, 12, 24, 36, 32, and 44) across buccal, lingual, and proximal surfaces. The criteria for the PI are 0 (no plaque), 1 (plaque detectable by the probe), 2 (moderate accumulation visible to the naked eye), and 3 (abundant soft deposits). Dental hygiene was categorized as extremely good, good, less than good, and extremely poor [8].

Saliva Sample Collection and Analysis

Participants were given instructions to abstain from oral hygiene, eating, or smoking for one hour before the collection process. Saliva was collected using paraffin wax pellets (Ivoclar Vivadent AG, Liechtenstein) to stimulate saliva production, which was expectorated into sterile 50-ml Corning Falcon Conical Centrifuge Tubes (Corning Incorporated, Corning, NY, USA) until 5 ml was obtained. Samples were labeled and transported on ice to the Genetic Laboratory at Zheen International Hospital and stored at −20°C until analysis [9].

Genomic DNA was extracted using the PureLink™ Genomic DNA Mini Kit (Thermo Fisher Scientific Inc., Waltham, MA, USA), following the manufacturer's protocol with minor modifications. The steps included incubation with proteinase K and RNase A, lysis with genomic binding buffer, and ethanol precipitation. The lysate was transferred to a PureLink™ Spin Column (Thermo Fisher Scientific Inc., Waltham, MA, USA), washed with ethanol-based buffers, and eluted with a PureLink™ Genomic Elution Buffer (Thermo Fisher Scientific Inc., Waltham, MA, USA). The concentration and purity of DNA were evaluated using a NanoDrop instrument (Thermo Fisher Scientific Inc., Waltham, MA, USA), with acceptable results being more than 10 ng/μl and specified ratio values (260/280 value of 1.8 and 260/230 value of 2-2.2).

Quantitative detection of SM and LB spp. was performed using real-time PCR on a CFX96 system (Bio-Rad Laboratories, Inc., Hercules, CA, USA). Each reaction mixture contained 12.5 μl qMAXSen™ GREENqPCR Master Mix (Canvax, Germany), 5 μl of extracted DNA, 2 μl of each primer, and 3.5 μl nuclease-free water. The thermocycling protocol included an initial activation at 94°C for 10 minutes, followed by 45 cycles of denaturation at 95°C for 45 seconds, annealing (64°C for SM and 62°C for LB spp.) for 45 seconds, extension at 72°C for 45 seconds, and a final extension at 72°C for three minutes. Results were expressed as cycle threshold (Ct) values and converted to colony-forming units (CFU)/ml using standard curves [10].

**Figure 1:**
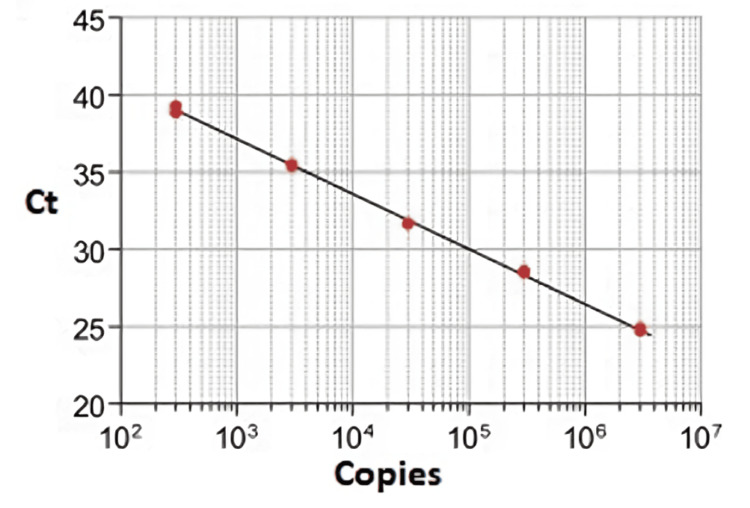
Standard curve indicating Ct values vs. SM and LB spp. numbers (CFU/ml). Serial 10 fold dilutions of SM and LB spp. DNA samples (ranging from 102 to 107 CFU/ml) were prepared to make a standard curve Ct: cycle threshold, SM: *Streptococcus mutans*, LB: *Lactobacillus​​​​​​​*, CFU: colony-forming units​​​​​​​, DNA​​​​​​​: deoxyribonucleic acid​​​​​​​

To establish a 10-fold dilution series ranging from 0.1 log10 to 7 log10 CFU equivalent, genomic DNA extracted from various types of strains was employed. As a negative control, 17.5 μl of double deionized water was utilized. By plotting the Ct against the logarithm of bacterial quantity (log10 CFU equivalent) for each run, standard curves were generated for each strain variant. The Ct value denotes the count of PCR cycles required to attain the threshold fluorescence level (Figure 1).

Primers were optimized by conventional PCR and agarose gel electrophoresis. The sequences and temperatures for annealing are provided in Table 1.

**Table 1:** Primer sequences and optimal annealing temperature

Primer name	Sequence (5^, ^to 3)	Optimal annealing temperature	Reference
Sm F5 Primer	5′-AGCCATGCGCAATCAACAGGT-3′	64°C	[11]
Sm R4 Primer	5′-CGCAACGCGAACATCTTGATCAG-3′		
LactoF Primer	5′-TGGAAACAGRTGCTAATACCG-3′	62°C	[12]
LactoR Primer	5′-GTCCATTGTGGAAGATTCCC-3′		

Ethical considerations

The study was conducted in compliance with the World Medical Association's Declaration of Helsinki, following ethical approval from the Ethical Review Committee of the College of Dentistry at Hawler Medical University (approval number: 552024). Participants were informed about the study's purpose and procedures and provided written consent before data collection began. Data confidentiality and participant privacy were strictly maintained throughout the study.

Statistical analysis

Data were analyzed using SPSS Statistics version 26 (IBM Corp. Released 2019. IBM SPSS Statistics for Windows, Version 26.0. Armonk, NY: IBM Corp.). The Chi-square test was used to compare categorical variables between the groups. The Mann-Whitney test was applied for continuous variables where the data distribution was not normal. p-values of ≤0.05 were considered statistically significant.

## Results

This study involved 100 participants, ranging in age from 18 to 30 years, with a mean age of 23.96 ± 3.64 years. Half of the participants (50%) were using fixed orthodontic appliances, while the other half served as the control group. The groups were matched for age and sex, with the majority being female 79 (79%) and the largest age group being 26-30 years 38 (38%) (Table 2).

**Table 2:** Age and sex distribution of the participants * calculated by Chi-square test

Variables		Fixed orthodontic appliance no. (%)	Control no. (%)	Total no. (%)	p-value*
Age	18-21	16 (32.0)	16 (32.0)	32 (32.0)	1.000
	22-25	15 (30.0)	15 (30.0)	30 (30.0)	
	26-30	19 (38.0)	19 (38.0)	38 (38.0)	
Sex	Male	11 (22.0)	10 (20.00	21 (21.0)	1.000
	Female	39 (78.0)	40 (80.0)	79 (79.0)	

In an examination of daily oral hygiene practices, 75 (75%) participants reported brushing their teeth daily, while the remainder brushed less frequently (p=0.488). Among those who brushed daily, 44 (58.7%) participants brushed twice a day, although this difference was not statistically significant across groups (p=0.302). All participants in the control group used traditional toothbrushes with toothpaste, in contrast to 45 (90%) participants in the orthodontic group. Additionally, five (10%) orthodontic group participants employed interdental brushes, as opposed to none in the control group (p=0.056). The study also explored the use of dental floss, revealing that 56 (56%) participants rarely or never used floss, with no significant difference between groups (p=0.081). Furthermore, 64 (64%) participants rinsed their mouths with water after meals, which also did not differ significantly between groups (p=0.680). Regular dental examinations were conducted by nine (18%) participants in the orthodontic group and 20 (40%) participants in the control group (p=0.015). Half of the orthodontic group (50%) avoided fluoride use, compared to 13 individuals (26%) in the control group (p=0.003). No significant difference was observed in the number of sugary meals consumed per day between the two groups (p=0.167), as presented in Table 3.

**Table 3:** Oral hygienic practices, fluoride, and dietary habits of the study groups * calculated by Chi-square test, ** calculated by Fisher’s exact test

Oral hygiene practices and dietary habits	Fixed orthodontic appliance no. (%)	Control no. (%)	Total no. (%)	p-value
How often do you brush your teeth?				
I brush them but not every day	14 (28.0)	11 (22.0)	25 (25.0)	0.488*
I brush them every day	36 (72.0)	39 (78.0)	75 (75.0)	
Tooth brushing frequency				
Once daily	16 (44.4)	11 (28.2)	27 (36.0)	0.302**
Twice daily	18 (50.0)	26 (66.6)	44 (58.7)	
Three times per day	2 (5.6)	1 (2.6)	3 (4.0)	
More than three times	0 (0.0)	1 (2.6)	1 (1.3)	
Method of cleaning the teeth				
Traditional toothbrush with toothpaste	45 (90.0)	50 (100.0)	95 (95.0)	0.056**
Interdental brush	5 (10.0)	0 (0.0)	5 (5.0)	
Use of dental floss				
Rarely or never	34 (68.0)	22 (44.0)	56 (56.0)	0.081**
Infrequently (once every few weeks)	7 (14.0)	11 (22.0)	18 (18.0)	
Every few days	4 (8.0)	12 (24.0)	16 (16.0)	
At least once a day	1 (2.0)	1 (2.0)	2 (2.0)	
Frequently (almost every day)	4 (8.0)	4 (8.0)	8 (8.0)	
Rinsing the mouth after meals				
Yes	34 (68.0)	30 (60.0)	64 (64.0)	0.680**
No	13 (26.0)	17 (34.0)	30 (30.0)	
Sometimes	3 (6.0)	3 (6.0)	6 (6.0)	
Do you go for regular dental checkups?				
Yes	9 (18.0)	20 (40.0)	29 (29.0)	0.015*
No	41 (82.0)	30 (60.0)	71 (71.0)	
Fluoride program				
Additional F measures, infrequently	1 (2.0)	10 (20.0)	11 (11.0)	0.003*
Fluoride toothpaste only	24 (48.0)	27 (54.0)	51 (51.0)	
Avoiding fluorides, no fluoride	25 (50.0)	13 (26.0)	38 (38.0)	
Dietary sugar frequency				
Maximum three meals per day (including snacks)	28 (56.0)	20 (40.0)	48 (48.0)	0.167**
Maximum five meals per day	14 (28.0)	24 (48.0)	38 (38.0)	
Maximum seven meals per day	5 (10.0)	5 (10.0)	10 (10.0)	
More than seven meals per day	3 (6.0)	1 (2.0)	4 (4.0)	
Total	50 (100.0)	50 (100.0)	100 (100.0)	

Results showed that 24% and 22% of the orthodontic group had moderate and high amounts of LB, respectively, compared with 8% and 2% of the control group, respectively (p<0.001). Same for SM levels in saliva, where 60% of the orthodontic group had low (16%), high (22%), or very high (22%) amounts, compared with 10% of the control group (p<0.001). Regarding the plaque amount, there was no significant difference between the groups (p=0.262), but it is evident that 62% of the orthodontic group had less than good oral hygiene, compared with 48% of the control group. No significant difference was detected between the groups regarding the saliva secretion rates (p=0.358), as presented in Table 4.

**Table 4:** Caries-related factors * calculated by Fisher’s exact test ** LB amounts (levels): 0 = very low (≤103 CFU/ml), 1 = low (104-105 CFU/ml), 2 = moderate (105-106 CFU/ml), 3 = high (≥106 CFU/ml) *** SM amounts (levels): 0 = very low (≤20,000 CFU/ml saliva), 1 = low (20,000-1,00,000 CFU/ml saliva), 2 = high (>1,00,000-1 million CFU/ml saliva), 3 = very high (>1 million CFU/ml saliva) LB: *Lactobacillus*, SM: *Streptococcus mutans*, CFU: colony-forming units

Caries-related factors	Fixed orthodontic appliance no. (%)	Control no. (%)	Total no. (%)	p-value*
LB amounts (levels)**				
Zero or very low amount	19 (38.0)	44 (88.0)	63 (63.0)	<0.001
Low amount	8 (16.0)	1 (2.0)	9 (9.0)	
Moderate amount	12 (24.0)	4 (8.0)	16 (16.0)	
High amount	11 (22.0)	1 (2.0)	12 (12.0)	
SM amounts (levels) in saliva***				
Zero or very low amount	20 (40.0)	45 (90.0)	65 (65.0)	<0.001
Low amount	8 (16.0)	1 (2.0)	9 (9.0)	
High amount	11 (22.0)	3 (6.0)	14 (14.0)	
Very high amount	11 (22.0)	1 (2.0)	12 (12.0)	
Plaque amount				
Extremely good oral hygiene (<0.4)	1 (2.0)	0 (0.0)	1 (1.0)	0.262
Good oral hygiene (0.4-1.0)	17 (34.0)	23 (46.0)	40 (40.0)	
Less than good oral hygiene (1.1-2.0)	31 (62.0)	24 (48.0)	55 (55.0)	
Poor oral hygiene (>2.0)	1 (2.0)	3 (6.0)	4 (4.0)	
Saliva secretion scores				
Normal saliva secretion	17 (34.0)	21 (42.0)	38 (38.0)	0.358
Reduced (0.9-1.1 ml saliva/min)	16 (32.0)	9 (18.0)	25 (25.0)	
Low (0.5-0.9 ml saliva/min)	14 (28.0)	14 (28.0)	28 (28.0)	
Very low, xerostomia (<0.5 ml saliva/min)	3 (6.0)	6 (12.0)	9 (9.0)	
Total	50 (100.0)	50 (100.0)	100 (100.0)	

No significant disparities were found between the two research groups in terms of the average rankings of the caries indicators: DMFT (p=0.790), DMFS (p=0.609), and PI (p=0.203), as shown in Table 5.

**Table 5:** Caries indicators of the study groups * calculated by Mann-Whitney test DMFT: decayed, missing, and filled teeth, DMFS: decayed, missing, and filled surfaces, PI: plaque index, SD: standard deviation

	Fixed orthodontic appliance			Control			
	Mean (SD)	Median	Mean rank	Mean (SD)	Median	Mean rank	p-value*
DMFT	5.30 (3.58)	5.00	51.27	5.18 (3.85)	5.00	49.73	0.790
DMFS	11.68 (12.40)	7.00	51.98	10.16 (11.12)	8.00	49.02	0.609
PI	1.26 (0.40)	1.26	54.19	1.19 (0.42)	1.11	46.81	0.203

In the orthodontic group, no significant correlations were found between practice scores and the levels of LB (p=0.298) and SM in saliva (Table 6).

**Table 6:** Cariogenic bacteria levels by practice scores in fixed orthodontic group * calculated by Fisher’s exact test LB: *Lactobacillus*, SM: *Streptococcus mutans*

Cariogenic bacteria levels	Poor practice (1-7) no. (%)	Good practice (8-14) no. (%)		Total no. (%)	p-value*
LB amounts (levels)					
Zero or very low amount	9 (47.4)	10 (32.3)	19 (38.0)		0.298
Low amount	2 (10.5)	6 (19.4)	8 (16.0)		
Moderate amount	6 (31.6)	6 (19.4)	12 (24.0)		
High amount	2 (10.5)	9 (29.0)	11 (22.0)		
SM amounts (levels) in saliva					
Zero or very low amount	9 (47.4)	11 (35.5)	20 (40.0)		0.674
Low amount	2 (10.5)	6 (19.4)	8 (16.0)		
High amount	3 (15.8)	8 (25.8)	11 (22.0)		
Very high amount	5 (26.3)	6 (19.4)	11 (22.0)		
Total	19 (100.0)	31 (100.0)	50 (100.0)		

Similarly, in the control group, there were no significant correlations between LB levels (p=0.521) and SM levels (p=0.157), as shown in Table 7.

**Table 7:** Cariogenic bacteria levels by practice scores in control group * calculated by Fisher’s exact test LB: *Lactobacillus*, SM: *Streptococcus mutans*

Cariogenic bacteria levels	Poor practice (1-7) no. (%)		Good practice (8-14) no. (%)		Total no. (%)	p-value*
LB amounts (levels)						
Zero or very low amount	12 (85.7)		32 (88.9)	44 (88.0)		0.521
Low amount	0 (0.0)		1 (2.8)	1 (2.0)		
Moderate amount	1 (7.1)		3 (8.3)	4 (8.0)		
High amount	1 (7.1)		0 (0.0)	1 (2.0)		
SM amounts (levels) in saliva						
Zero or very low amount		11 (78.6)	34 (94.4)	45 (90.0)		0.157
Low amount		1 (7.1)	0 (0.0)	1 (2.0)		
High amount		2 (14.3)	1 (2.8)	3 (6.0)		
Very high amount		0 (0.0)	1 (2.8)	1 (2.0)		
Total		14 (100.0)	36 (100.0)	50 (100.0)		

## Discussion

There is considerable evidence in the literature demonstrating that the presence of fixed orthodontic appliances in the oral cavity of dental patients could influence changes in oral microbial profiles. Following the application of orthodontic appliances, the structure, metabolism, and composition of dental plaque would change, leading to a general increase in the levels of the microbial population, particularly *Streptococcus* and *Lactobacillus* [5].

SM has been firmly linked to the start of dental caries, although studies suggest that LB may be more important in the advancement of caries lesions and has been identified to be the cause of a small fraction of coronal caries [2].

In the current study, the frequency and levels of factors associated with dental caries, including LB bacteria and SM, as well as plaque accumulation and salivary secretion, were examined among patients using fixed orthodontic appliances compared to the control group. The investigation revealed that the quantity and prevalence of LB and SM were higher in patients with fixed orthodontics than in the control group. The assessment of plaque in both groups indicated that most individuals had less than good oral hygiene. The levels of LB and SM did not differ significantly between individuals with good and poor oral hygiene practices within both the fixed orthodontic group and the control group. Oral hygiene practices, including daily tooth brushing and dental flossing, were performed in both groups, albeit flossing was less frequent. Sugar consumption occurred daily in both groups. Dental checkups were more frequent in the control group. The fluoride therapy index indicated that the control group had better status and used fluoride more frequently.

The placement of fixed orthodontic appliances can lead to the accumulation and adherence of bacterial biofilm, potentially causing demineralization of the enamel and gingival inflammation [2,13]. Consequently, it is crucial that each patient utilizing orthodontics initially and throughout the treatment receives adequate and appropriate oral hygiene education, as non-compliance can pose significant challenges [14]. In this study, the mean age of participants was 23.96 ± 3.64 years, and most participants in both groups were female, similar to the findings of Muñoz et al. [15] in Colombia, where the impactful bacteria SM on dental caries in patients with and without fixed orthodontic appliances were studied. The mean age in the Muñoz et al. study was 20 years, which was younger than that in the current study, attributed to a smaller sample size and different study methodologies. The sex distribution was similar between the two studies and also in another study by Mummolo et al. [16].

Patients with fixed orthodontic appliances and healthy individuals did not differ significantly in terms of the frequency of tooth brushing and the method used, with most individuals in both groups practicing daily tooth brushing. This aligns with a study in Iran by Khanjani et al. [17], which found no significant differences in oral hygiene practices among participants, similar to another study in Jordan by Alhaija et al. [18]. However, a study in France showed significant differences in oral hygiene practices between patients with a history of periodontitis and healthy individuals, with the periodontitis group practicing more intensive hygiene measures [19].

The use of dental floss was infrequently reported in both groups, with no significant differences observed, contrasting with a study by Santos et al. [20] that explored the impact of telemedicine on adherence to oral hygiene practices, including flossing. An observational before-and-after study in Croatia also noted significant differences in floss usage pre- and post-intervention, likely due to effective information dissemination and education in certain communities [21]. Dental checkups and consultations among participants in both groups were irregular, with the control group visiting dentists more frequently. This finding contrasts with other studies where regular dental visits were more common, likely due to greater accessibility to facilities and higher awareness among participants [21]. Fluoride use was also examined, revealing that healthy individuals used fluoride more frequently than the fixed orthodontic appliance group. In contrast, a study in Thailand by Thanetchaloempong et al. [1] reported that all participants used fluoride for dental care, reflecting higher adherence compared to the current study's findings. Also, in a clinical trial study in Sweden, there was a significant difference in the use of mouthwash and fluoride among the study participants [22]. The present study has indicated that levels of LB and SM bacteria are higher in patients with fixed orthodontic appliances compared to a healthy control group. This finding aligns with a study by Al‐Melh et al. [5], which focused on the prevalence and quantities of caries-related bacteria in patients with orthodontic brackets. The study revealed that the colonization of LB and SM increased following the application of fixed orthodontic appliances, correlating closely with heightened dental caries risk. Further studies, including those conducted in Italy [16] and India [23], have consistently demonstrated that fixed orthodontic treatment alters the oral environment, enhancing plaque formation and microbial colonization, thereby increasing the levels of decay-causing bacteria and associated risks of dental caries. Similarly, research from Kurdistan, Iraq, supports these findings, showing a correlation between increased levels of these bacteria and higher caries incidence [2]. An investigation into plaque levels among the two groups indicated that most participants maintained good oral hygiene. In a study by Cenzato et al. [24], which examined the effects of fixed orthodontic appliance removal on oral hygiene, it was discovered that there was a notable difference in plaque accumulation among the participants. Specifically, individuals who had utilized fixed orthodontic appliances exhibited a greater mean plaque amount than those who had not. A parallel study in Iran confirmed these results, highlighting a significant increase in plaque accumulation among patients with fixed appliances [25].

Regarding saliva secretion, studies by Dallel et al. [26] in Tunisia and Wang et al. [27] found that fixed orthodontic appliances did not significantly alter saliva production compared to healthy controls. Wang's study, however, suggested that increased saliva secretion linked to orthodontic treatment could potentially contribute to the proliferation of cariogenic bacteria. Despite the elevated bacterial levels in fixed orthodontic patients, no significant differences were observed in the decay indices (DMFT, DMFS, PI) between fixed orthodontic and healthy individuals. This was contrary to a study in South Korea, which indicated higher decay indices among orthodontic patients [28]. However, a long-term study over 30 years in Australia by Doğramacı and Brennan [29] found that fixed orthodontic treatment did not increase the risk of dental decay.

The findings of this study suggest that the presence of fixed orthodontic appliances significantly alters the oral microbial environment, notably increasing the levels of cariogenic bacteria such as LB and SM. These findings could have important implications for developing future orthodontic treatment protocols. Orthodontic care providers must integrate comprehensive oral hygiene education and preventive care strategies into treatment plans. Regularly monitoring bacterial levels could become a standard practice, allowing for timely interventions to prevent demineralization and caries development. Incorporating adjunctive tools such as interdental brushes and fluoride treatments may enhance the effectiveness of oral hygiene measures. Additionally, patient-specific risk assessments for caries should be considered when planning orthodontic interventions to minimize the adverse effects associated with increased bacterial colonization.

The limitation of this study was that we investigated only a few bacterial species of importance in caries development and progression. With the increasing utility of NextGen sequencing technologies that reveal the total microbiota of the samples in question, several novel bacterial species whose role in caries was hitherto unknown have been uncovered during recent years. Sequencing techniques like 16S rRNA gene metagenomics would have provided a comprehensive delineation of the microbiota in the dental plaque retained around the orthodontic braces. Additionally, while this study did not directly explore the potential correlations between cariogenic bacteria levels and various oral hygiene and dietary factors within each group, such an analysis could provide valuable insights into specific behaviors. Future studies should consider a more granular examination of these correlations.

## Conclusions

The current study showed that patients with fixed orthodontics exhibited higher levels of LB and SM compared to healthy controls. Despite these elevated bacterial counts, there was no significant difference in decay indices between the two groups. This outcome suggests that the high baseline levels of these indices in both groups might obscure potential differences. These findings underscore the necessity of stringent oral hygiene and regular monitoring for patients undergoing fixed orthodontic treatment to mitigate potential increases in caries risk.
